# Supplementary material for: Causal Relationship between Aging and Anorexia Nervosa: A White-Matter-Microstructure-Mediated Mendelian Randomization Analysis
Source: Biomedicines. 2024 Aug 16;12(8):1874. doi: 10.3390/biomedicines12081874 (PMC11351342; doi:10.3390/biomedicines12081874)
Supplement: Supplementary file 1 [file biomedicines-12-01874-s001.zip › Supplementary Figure/Figure S8/Leaveoneout plot of MR analysis for TL to ubm-b-1518.pdf]

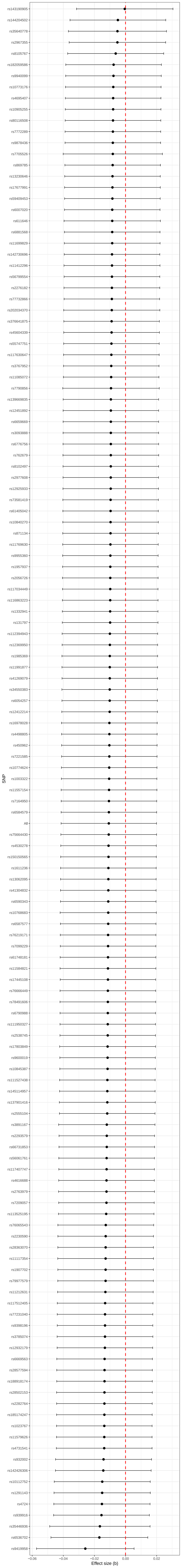

rs143190905
rs144204502
rs35640778
rs2967355
rs8105767
rs182059586
rs9940099
rs10773176
rs4695407
rs10905255
rs80116508
rs7772289
rs9878436
rs7705526
rs869785
rs13230646
rs17677991
rs59409453
rs6007020
rs611646
rs6881568
rs11699829
rs142730696
rs11412296
rs56799554
rs2276182
rs77732866
rs202034370
rs376641875
rs45604339
rs55747751
rs117630647
rs3767952
rs11085072
rs7790856
rs139669835
rs12451892
rs6659669
rs3093888
rs6776756
rs762679
rs8102497
rs2977608
rs12925933
rs73581419
rs61405042
rs10840270
rs871134
rs11769630
rs9955360
rs1957937
rs2056726
rs117034449
rs116863223
rs1332941
rs131797
rs112394943
rs12369950
rs1985369
rs11991877
rs41269079
rs34550383
rs6054257
rs12412214
rs16978028
rs4498805
rs450962
rs7221585
rs10774624
rs1003322
rs11557154
rs7164950
rs6584579
All
rs75664430
rs4530278
rs150150565
rs1611236
rs13062095
rs41304832
rs6590343
rs10768683
rs6587577
rs76219171
rs7099229
rs61748181
rs11584821
rs17445108
rs76666449
rs78491606
rs6790988
rs111950327
rs2538745
rs17803849
rs9600019
rs10845387
rs111527438
rs145114957
rs137901416
rs2555104
rs3891167
rs2293579
rs66731853
rs56061761
rs117407747
rs4616688
rs2763979
rs7209057
rs113525195
rs76065543
rs2230590
rs28363070
rs11117354
rs1907702
rs79977579
rs11212631
rs117512405
rs77231040
rs9398196
rs3785074
rs12932179
rs6669563
rs28577594
rs188918174
rs28502153
rs2282764
rs185174247
rs1023767
rs11579626
rs4731541
rs932002
rs142426306
rs10112752
rs1291143
rs4724
rs939916
rs35446936
rs6536702
rs9419958
SNP
−0.06
−0.04
−0.02
0.00
0.02
Effect size (b)
